# Professor Bartlett’s Address

**Published:** 1850-01

**Authors:** 


PROFESSOR BARTLETT'S ADDRESS.

The beautiful and appropriate tribute rendered to Dr. Wells, author of the "Essay on Dew," by Prof. Bartlett, cannot fail to please those who are fond of all that is excellent and becoming in the human character. The English language boasts of no philosophical treatise more perfect in all its details, than the "Essay on Dew," and there are few Americans who read the tributes of admiration, bestowed in European scientific works on that celebrated essay, who are aware of the fact that its author was an American. He studied medicine under the accomplished Dr. Garden, a physician, a scholar, and philosopher. Dr. G. was a favorite of the great Linnæus, and the fragrant plants, called Cape Jessamines, were named Gardenia radicans and grandiflora, in his honor. While breathing the rich perfumes of these flowers, which exhale their sweetness in great power, when the dew is on them, how little did he expect that his young student was to lift t e veil, and explain, with perfectness, the whole of those phenomena, which the master had often looked upon through the eyes of Nature's great interpreter, when he says:

> "And that same dew, which sometime on the buds
> Was wont to swell like round and orient pearls,
> Stood now within the pretty flow'ret's eyes."

Dr. Wells is worthy of a noble tribute, and his memory and character have received it at the hands of Professor Bartlett.

